# The pentose phosphate pathway in industrially relevant fungi: crucial insights for bioprocessing

**DOI:** 10.1007/s00253-021-11314-x

**Published:** 2021-05-05

**Authors:** Audrey Masi, Robert L. Mach, Astrid R. Mach-Aigner

**Affiliations:** 1 Christian Doppler Laboratory for Optimized Expression of Carbohydrate-Active Enzymes, Institute of Chemical, Environmental and Bioscience Engineering, TU Wien, Gumpendorfer Str. 1a, A-1060 Vienna, Austria; 2 Institute of Chemical, Environmental and Bioscience Engineering, TU Wien, Gumpendorfer Str. 1a, A-1060 Vienna, Austria

**Keywords:** Pentose phosphate pathway, Industrially relevant fungi, Yeast, Filamentous fungi

## Abstract

**Abstract:**

The pentose phosphate pathway (PPP) is one of the most targeted pathways in metabolic engineering. This pathway is the primary source of NADPH, and it contributes in fungi to the production of many compounds of interest such as polyols, biofuels, carotenoids, or antibiotics. However, the regulatory mechanisms of the PPP are still not fully known. This review provides an insight into the current comprehension of the PPP in fungi and the limitations of this current understanding. It highlights how this knowledge contributes to targeted engineering of the PPP and thus to better performance of industrially used fungal strains.

**Key points:**

*• Type of carbon and nitrogen source as well as oxidative stress influence the PPP.*

*• A complex network of transcription factors regulates the PPP.*

*• Improved understanding of the PPP will allow to increase yields of bioprocesses.*

## Introduction

In the early 1920s, Otto Warburg, a German Nobel laureate, investigated the mechanism of oxygen consumption in animal cells. His work was the beginning of the discovery of the pentose phosphate pathway (PPP). In the 1930s, he discovered the first flavoprotein and a non-protein component that is essential for the enzyme activity and acts as an electron carrier: the diphosphopyridine nucleotide DPN (now called NAD+ (nicotinamide adenine dinucleotide)) (Warburg 1932; Warburg and Christian 1936). He studied the oxidation of glucose-6-phosphate (G6P) to 6-phosphogluconate (6PG) by an enzyme, which he called *Zwischenferment* (ZWF1) or intermediate enzyme and is now called glucose-6-phosphate dehydrogenase (G6PDH). He found a second co-enzyme that he named TPN (triphosphopyridine nucleotide, now known as NADP+ (nicotinamide adenine dinucleotide phosphate)). The discovery of the requirement of TPN for the oxidation of G6P and also for the oxidation of 6PG prompted Warburg and his team to propose the existence of a “direct oxidative pathway” (Horecker 2002). They formulated the hypothesis that this pathway would be an alternative to glycolysis and is involved in cellular respiration. Franck Dickens and Fritz Lipmann, among other biochemists, who investigated cell respiration, shared this view (Horecker 2002).

This work was pursued in the USA by a member of Warburg’s laboratory, Erwin Haas, joined by Bernard Horecker. During the 1950s, the work of Horecker’s group allowed to elucidate the PPP and to propose a first description of it in animals (Gunsalus et al. 1955). This significant progress was made possible by the development of analytical methods, such as ion chromatography, as well as by discoveries made by other research teams including the teams of Dickens, Racker, Cohen, Lampen, Dickens, and Ashwell (Dickens 1938; Dickens and Glock 1951; Dickens and Williamson 1956; Horecker 2002).

**Fig. 1:**
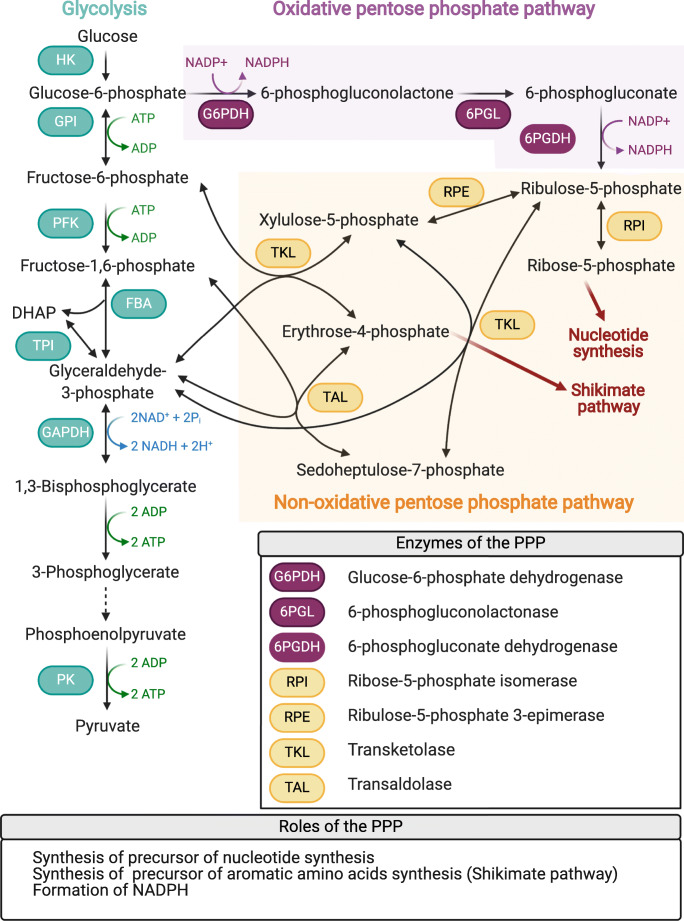
Schematic drawing of the pentose phosphate pathway, its enzymes, and its connection to glycolysis. Single arrows indicate a non-reversible reaction, and double arrows indicate a reversible reaction. The oxidative pentose phosphate pathway and the non-oxidative pentose phosphate pathway are identified by purple or yellow background, respectively. Red arrows indicate links to other biochemical pathways. Abbreviations of glycolysis enzymes: HK, hexokinase; GPI, glucose-6-phosphate isomerase; PFK, phosphofructokinase 1; FBA, fructose-bisphosphate aldolase; TPI, triosephosphate isomerase; GAPDH, glyceraldehyde-3-phosphate dehydrogenase; PK, pyruvate kinase (Stincone et al. 2015)

In the 1960s and 1970s, the first research groups started investigating the regulation of the PPP in yeasts and filamentous fungi. The interest in the PPP in fungi increased at the end of the 1990s with the development of metabolic engineering for industrial biotechnology. Due to its central role in carbon metabolism, the PPP became a critical target to increase the production of molecules of interest in industrially used fungal strains (Stincone et al. 2015). Since then, the PPP was investigated in many organisms and was shown to be an ubiquitous pathway present in every eukaryote and most prokaryotes. Figure 1 presents the current understanding of the pathway**.**

With the exception of archaea, the currently known structure of the PPP is conserved among most prokaryotes and eukaryotes*.* However, even if archaea have a different pathway organization, it includes similar reactions suggesting a very ancient origin of the PPP in evolution (Bräsen et al. 2014)*.* Table 1 summarizes the enzymes associated with the PPP in different organisms.

**Table 1:** Presence of the PPP enzymes among the three domains (Soderberg 2005; Oost and van der Siebers 2007)

Enzyme	Abbr.	EC no.	Present in archaea	Present in bacteria	Present in eukarya
Phosphohexulose isomerase	PHI	5.3.1.27	Except for some halophiles and Thermoplasmatales	Methylotrophs in particular	/
Hexulose phosphate synthase	HPS	4.1.2.43	Except for some halophiles and Thermoplasmatales	Methylotrophs in particular	/
Glucose-6-phosphate dehydrogenase	G6PDH	1.1.1.49	Haloarchaea	✓	✓
6-phosphogluconolactonase	6PGL	3.1.1.31	⨯	⨯	✓
6-phosphogluconate dehydrogenase	6PGDH	1.1.1.44	⨯	✓	✓
Ribose-5-phosphate isomerase	RPI	5.3.1.6	✓	✓	✓
Ribulose-5-phosphate 3-epimerase	RPE	5.1.3.1	*Methanococcus* spp., Thermoplasmatales, not in most other archaea	✓	✓
Transketolase	TKL	2.2.1.1	*Mca. jannaschii*, Thermoplasmatales, Thermococcales, Sulfolobales, Thermoproteales, not in most haloarchaea, other methanogens and Archaeglobales		✓
Transaldolase	TAL	2.2.1.2	*Methanococcus* spp., Thermoplasmatales	✓	✓

Most archaea do not have enzymes for the oxidative pentose phosphate pathway (OPPP) except orthologs of the 6-phosphogluconate dehydrogenase (6PGDH), which were identified mainly in halophilic archaea. And just a few archaea have a complete non-oxidative pentose phosphate pathway (NOPPP) (Soderberg 2005; Oost and van der Siebers 2007). *Methanococci* pose an exception here: their non-oxidative phase is similar to the standard PPP; however, they do not have any of the enzymes of the oxidative phase. The hypothesis is that they do not require the oxidative phase for the synthesis of ribose-5-phosphate (R5P) because it is formed through the reversible reactions of the non-oxidative phase (Soderberg 2005; Oost and van der Siebers 2007). The conservation of the NOPPP among archaea suggests an ancient evolutionary origin of the NOPPP, while the OPPP seems to arise more recent in evolution.

The oxidative phase comprises three irreversible reactions, while the non-oxidative phase is composed of reversible reactions only. The oxidative phase starts with the oxidation of the G6P, a substrate generated during glycolysis. Thus, this first reaction is a metabolic crossroad between the glycolysis and the PPP. The results of the oxidative phase are two molecules of NADPH and ribulose-5-phosphate (Ru5P). Ru5P then enters the non-oxidative phase of the PPP. In the non-oxidative phase, the cells need to control the number of reactions and their orientation. The metabolites involved in this phase, either as substrates or products, are also involved in other pathways. This connection to other pathways assigns a central role in metabolism to the NOPPP. For example, glyceraldehyde-3-phosphate (G3P) and fructose-6-phosphate (F6P) are intermediate products of the glycolysis and linked to the NOPPP, and erythrose-4-phosphate (E4P) is a product of the NOPPP and at the same time a precursor for the shikimate pathway. The latter links carbon metabolism with the biosynthesis of aromatic compounds such as phenylalanine, tryptophan, and tyrosine. The PPP also leads to the production of R5P, which serves as a precursor for the synthesis of nucleotides. These strong connections between the NOPPP and other biochemical pathways might require a dynamic regulation of the involved enzymes (Stincone et al. 2015).

Summarizing, the PPP has three main functions: (i) increasing the reducing power of the cell through NADPH production; (ii) the production of R5P, which is a precursor for nucleotide biosynthesis; and (iii) synthesis of E4P, a precursor for aromatic amino acid biosynthesis. The PPP also allows the synthesis and degradation of four-, five-, and seven-carbon sugars. Considering such a central role in carbon metabolism, the PPP unsurprisingly is one of the most targeted pathways in metabolic engineering. Obviously, the understanding of the mentioned regulation of the PPP is highly beneficial for knowledge-based pathway engineering.

## Regulation of the PPP

Earlier studies reported a regulatory influence of carbon sources and nitrogen sources on the PPP and linked this to the NADPH production by the PPP. The role of the PPP as NADPH producer was also the primary explanation for its participation in the redox response. Nevertheless, recent studies have shown that the regulatory mechanisms are more complex than previously believed. Only the capacity of the PPP to produce NADPH is insufficient to explain its involvement in the redox response. Additionally, the regulatory mechanisms seem to operate at multiple levels, i.e., transcription, translation, enzyme activity, and metabolism.

### Regulation of the PPP in response to oxidative stress

The PPP produces NADPH during the OPPP and, therefore, is a significant source of NADPH for the cells. NADPH is a primary source of electrons in anabolic reactions, such as fatty acid biosynthesis, nucleotide biosynthesis, and glutathione reduction. Due to its involvement in reducing glutathione, NADPH is essential for resistance of cells to oxidative stress. Thus, the capacity of the PPP to generate NADPH was linked to a role in oxidative stress response (Bruinenberg et al. 1983). However, several findings from studies performed in *Saccharomyces cerevisiae* suggest that the role of PPP in oxidative stress response goes beyond NADPH generation only.

For example, strains deficient for enzymes of the NOPPP were sensitive to oxidants, although NADPH is only produced in the OPPP (Juhnke et al. 1996; Ralser et al. 2009). On the other hand, an accumulation of intermediates of the NOPPP was observed upon cell exposition to oxidants (Ralser et al. 2007; Ralser et al. 2009).

In another study, deletion strains were constructed in *S. cerevisiae* lacking the G6PDH (which is ZWF1 in *S. cerevisiae*) and/or one of the NOPPP enzymes, i.e., either the transketolase 1 (TKL1) or the ribulose-5-phosphate 3-epimerase 1 (RPE1) or the transaldolase 1 (TAL1) (Krüger et al. 2011). Strains lacking ZWF1 cannot use the PPP to reduce NADP+ because this is the first reaction of the PPP and non-reversible. Interestingly, among the double deletion strains, the one lacking TAL1 and ZWF1 was the only viable strain. This strain was exposed to oxidative stress generated via growth on agar plates containing 1 mM H_2_O_2_ and compared to single deletion strains of ZWF1 and TAL1. The single deletion strain of TAL1 was not sensitive to oxidative stress, the ZWF1 deletion strain was sensitive to oxidative stress, and the double deletion strain was more sensitive to oxidative stress than the ZWF1 single deletion strain. Further, the authors expressed a sedoheptulokinase (SHPK) to increase the flow through the PPP in the cell. SHPK is an enzyme, absent in yeasts, which converts sedoheptulose in sehoheptulose-7-phosphate (S7P), an intermediate of the NOPPP. Strains expressing the SHPK were more resistant to H_2_O_2_ exposition than the wild type, suggesting that the increase of the flux through the NOPPP increases resistance to oxidative stress. The investigation was continued by generating more strains bearing deletions of enzymes of the PPP and exposing the generated strains to three different oxidants: H_2_O_2_, diamide, and cumene-hydroperoxide (CHP). Strains with deletion of ZWF1 or RPE1 or TKL1 were more sensitive to the three tested oxidants than the wild type. A SOL4 deletion strain (deficient in 6-phosphogluconolactonase (6PGL)) was more sensitive to diamide but more resistant to CHP and H_2_O_2_, while a transketolase 2 (TKL2) deletion strain was more sensitive to H_2_O_2_ solely. TKL2 is a paralogue of TKL1 and arose from whole genome duplication. A TAL1 deletion strain was more sensitive to H_2_O_2_ but more resistant to diamide. A SOL3 deletion strain (deficient in 6PGL, *sol*3 and *sol*4 are two paralogous genes) had an increased resistance to each of the tested oxidants. The deletion strains were also exposed to other environmental stress conditions (NaCl for salt stress, sorbitol for osmotic stress, and MnCl_2_ for heavy metal stress) to see whether the response observed before was specific to oxidative stress. Interestingly, none of the mutants exhibited a stress phenotype. Finally, the authors measured the ratio of NADPH/NADP+ in cell-free extracts of the strains bearing deletion(s) of NOPPP enzyme(s) and compared it to the ratio in the wild-type exposed to oxidant stress. They found a similar ratio of NADPH/NADP+; consequently, this does not explain the changed response to the oxidants by the mutant strains (Krüger et al. 2011).

In the same study, LC-MS/MS (liquid chromatography coupled with tandem mass spectrometry) was used to quantify the PPP intermediates (i.e., 6PGL, R5P, and S7P) produced by the wild-type and the deletion strains exposed to H_2_O_2_. In the wild-type strain and in the SOL3 deletion strain, H_2_O_2_ treatment triggered no or only a small increase of the intermediates (below 10-fold; maximum concentration below 10 nmol·ml^−1^·OD600^−1^). In contrary, in the TAL1, RPE1, and TKL1 deletion strains, the concentrations of 6PG, R5P, and S7P increased strongly (up to 100-fold; concentrations up to 350 nmol·ml^−1^·OD600^−1^) (Krüger et al. 2011).

To understand how these changes are regulated, Krüger et al. investigated the mutant strain MR105. The MR105 strain carries a deletion of the endogenous triosephosphate isomerase (TPI) and expresses a mutant allele. In this strain, the TPI activity is just 30% compared to that of the wild type. It was shown that intermediates of the PPP are increased in MR105. This strain has also an increased resistance to oxidants and an increased NADPH/NADP+ ratio (Ralser et al. 2006; Ralser et al. 2007).

A transcriptome analysis was performed to compare changes in gene expression caused by the deficiencies of enzymes of the PPP or by H_2_O_2_ exposition (Krüger et al. 2011). The strain MR105 was used as a control as it has increased concentrations of intermediates of the PPP independent from oxidative stress. The transcriptomes of six conditions were analyzed, i.e., the MR105 strain, the wild-type strain, the wild-type strain treated with H_2_O_2_, and strains bearing deletion of TAL1, TKL1, or ZWF1, respectively. Transcriptomes of the strain MR105 and the wild-type treated with H_2_O_2_ shared 40% of the targeted genes, which amount for 140 transcripts. This result is surprising because H_2_O_2_ treatment creates an oxidative environment, whereas a decrease in TPI activity shifts the NADPH/NADP+ ratio towards a reductive environment (Ralser et al. 2007). Moreover, only 60 of the 140 transcripts were found to correlate with the redox state, i.e., had a different level in the MR105 strain compared to the H_2_O_2_-treated wild type. Thus, the authors proposed a regulation of the PPP due to the change in primary carbon metabolism rather than to the redox state (Krüger et al. 2011). Finally, the authors performed further investigations on transcript levels employing RT-qPCR and proteomics on targets belonging to the gene ontology categories of cellular respiration and chromatin assembly and disassembly. These measurements were performed with the wild-type and single deletion strains of TKL1, TAL1, or ZWF1. They observed a defect in timing and induction of gene expression during the stress response in the deletion strains. Thus, the regulation of gene expression during the oxidative stress response requires the activation of these enzymes (Krüger et al. 2011).

### Regulation of the PPP in response to nitrogen

Osmond and Rees first reported an influence of the nitrogen source on the activity of the PPP enzymes in *Candida utilis* (Osmond and Ap Rees 1969)*.* Nevertheless, their work did not provide any insight into the possible molecular regulatory mechanism. Some years later, Hankinson and Cove observed in *Aspergillus nidulans* an increased activity of the PPP enzymes when the fungus was grown in the presence of nitrate. Their work led to the first description of the PPP regulation by nitrate in filamentous fungi (Hankinson and Cove 1972; Hankinson 1974; Hankinson and Cove 1974).

#### Proposed mechanism in *C. utilis* by Osmond and Rees

After growth of the yeast with nitrate or yeast extract as nitrogen source, Osmond and Rees compared the activities of enzymes of the PPP and of glycolysis. The activities of two PPP enzymes, i.e., the G6PDH and the TKL, were 2.5-fold higher in the presence of nitrate. The other enzymes of the PPP showed a smaller increase in activity. Then, the yeast was pre-grown on a complex medium, starved from nitrogen, and separated in two subcultures; one was transferred to a complex medium containing nitrate, the other was transferred to the same medium supplemented with cycloheximide. After this transfer, the activity of the previously mentioned two enzymes increased; however, in the presence of cycloheximide, this was not observed. Further, it was shown that the cycloheximide effect is reversible. Cycloheximide inhibits protein synthesis by inhibiting the translational elongation suggesting that nitrate does not affect the enzyme activities directly but influences their biosynthesis. Unfortunately, Osmond and Rees did not suggest any molecular mechanism. They hypothesized that the need for NADPH regulates the PPP and argued that the formation of amino acids from nitrate requires NADPH, and the primary source of NADPH is the PPP. Thus, when grown on nitrate, yeasts would increase the activity of the G6PDH, this increased activity would lead to a higher flux through the PPP, and consequently, more NADPH would be produced.

In case of the TKL, Osmond and Rees speculated that the increased activity might avoid an accumulation of E4P, which would inhibit the glucose-6-phosphate isomerase (GPI), leading to an inhibition of the glycolysis (Osmond and Ap Rees 1969). However, no experimental proof supported this hypothesis, and the authors again did not provide any molecular regulatory mechanism. Hankinson and Cove, starting with a similar experiment, provided a breakthrough with the first hypothesis of the molecular regulation of the PPP by nitrogen (Hankinson and Cove 1972; Hankinson and Cove 1974).

#### Proposed mechanism in *A. nidulans* by Hankinson and Cove

Hankinson and Cove grew an *A. nidulans* wild-type strain in the presence of urea or urea and nitrate as nitrogen source. They observed a 2-fold higher activity of four enzymes of the PPP, i.e., the G6PDH, the 6PGDH, the TKL, and the TAL, and a 3-fold higher activity of the GPI compared to growth without nitrate. The ribose-5-phosphate isomerase (RPI) activity was not significantly affected in the presence of nitrate (Hankinson and Cove 1974).

Then, Hankinson and Cove performed the same experiment with mutants for the *nia*D gene (*nia*D*-*) and the *cnx* gene (*cnx*-). The *nia*D gene encodes a nitrate reductase, and *cnx* encodes a protein required for the synthesis of precursor Z, an intermediate of the molybdopterin cofactor pathway. The molybdopterin cofactor is required for the activity of a nitrate reductase. Consequently, mutations in the *nia*D locus or in any of the five *cnx* loci cause loss of nitrate reductase activity; hence, these mutants are unable to metabolize nitrate. They also generated a defective mutant for *nii*A (*nii*A-4), which is the nitrite reductase encoding gene, and a mutant for *nir*A (*nir*A^-^1). NirA positively controls the expression of *nia*D and *nii*A genes.

Surprisingly, in certain *nia*D- and *cnx*- mutants, nitrate increased the activities of PPP enzymes although the mutants cannot metabolize nitrate. Other mutants had high activities of these enzymes even in absence of nitrate. Due to these findings, it was concluded that neither nitrate nor nitrite can directly trigger the increased enzyme activity (Hankinson and Cove 1974). Further, in the *nir*A^-^1 mutants, nitrate did not increase the activity of the PPP enzymes. Other, the *nii*A-4 mutants responded to nitrate with increased activity of the PPP enzymes; however, they are only able to reduce nitrate to nitrite. Consequently, nitrate is not a direct inducer of the observed increase of the activity of PPP enzymes but a yet unknown factor that serves as mediator, which was called the *nir* product. These results also suggest that a modification of the concentration of NADP+/NADPH is not the cause of the increased activity of the PPP enzymes. Hankinson and Cove proposed the hypothesis that nitrate activates the expression of the *nir* product, which causes the increase in PPP enzyme activity: when the *nir* product is present in an active state, the activities of the PPP enzymes are high; when the *nir* product is present in an inactive state, these activities are low (Hankinson and Cove 1974).

The authors also addressed the point that the obtained results differ from the results reported in *C. utilis*. In *C. utilis* nitrate increased the activity of the G6PDH and the TKL, however, only to a very low extent the 6PGDH activity. Hankinson and Cove hypothesized that this difference can be explained by the reduced contribution of the malic enzyme, the pyridine nucleotide transhydrogenase, and the mannitol dehydrogenase to provide NADPH in *A. nidulans* under the tested growth conditions (Hankinson and Cove 1974). Based on the current knowledge (Horecker 2002), they assumed that the malic enzyme in yeasts might generate more NADPH during growth on glucose than in *A. nidulans.* In *A. nidulans* otherwise, the G6PDH and the 6PGDH might be the main source of NADPH; thus, if an increase of the NADPH concentration is needed, an increased activity of these two enzymes is necessary. A latter work from Hankinson and Cove confirmed that indeed also the mannitol dehydrogenase has a reduced contribution to NADPH generation in *A. nidulans* (Hankinson and Cove 1975).

#### From the hypothesis of Cove and co-workers to the current model

**Fig. 2:**
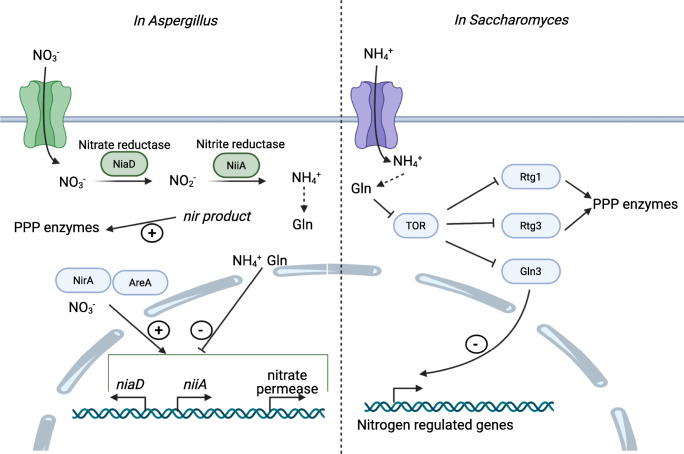
Schematic drawings of the proposed nitrogen regulation in *Aspergillu*s and *Saccharomyces*. In *Aspergillus* nitrate is reduced by consecutive action of two enzymes. Nitrate is reduced to nitrite by the nitrate reductase NiaD, and then nitrite is reduced to ammonium by the nitrite reductase NiiA, also called Nir. Ammonium is further metabolized to glutamine and glutamate. The *nir* product, which is currently not characterized, presumably activates the PPP enzymes through an unknown mechanism. Glutamine and ammonium repress the transcription of s *nia*D, of *nii*A, and of the nitrate permease-encoding gene. The transcription factors NirA and AreA as well as nitrate activate the transcription of the genes involved in nitrate metabolism (Soderberg 2005; Oost and van der Siebers 2007). In *Saccharomyces*, glutamine is produced from different sources, ammonium is one of them. When present, glutamine inhibits the TOR protein, which itself represses Rtg1, Rtg3, and Gln3 expression. When inhibited by TOR, Gln3 cannot translocate in the nucleus and act as a transcription activator of the nitrogen-regulated genes. Rtg1 and Rtg3 have been shown to have an impact on activity of the PPP enzymes through an unknown mechanism (Soderberg 2005; Oost and van der Siebers 2007). Consequently, in the presence of glutamine, TOR is not expressed resulting in active Rtg1 and Rtg3 as well as Gln3 and the latter to activation of nitrogen metabolism. A hypothesis to explain the effect of nitrate on the PPP enzymes in *Aspergillus* could be a mechanism similar to what has been observed in *Saccharomyces.* It can be hypothesized that the *nir* product is glutamine, and it impacts the PPP through a pathway involving TOR, Rtg1, Rtg3, and Gln3

The current understanding of nitrate regulation of the PPP is summarized and described in Fig. 2 (Hankinson and Cove 1974; Krappmann and Braus 2005; Han et al. 2016).

### Regulation of the PPP in response to the carbon source

Several studies reported an effect of the carbon source on the activities of the PPP enzymes; however, most studies propose that this effect is indirect (Bruinenberg et al. 1983; Witteveen et al. 1989; Peleato et al. 1991; David et al. 2005; David et al. 2006; Runquist et al. 2009; Khosravi et al. 2018). As mentioned above, the flux through the PPP might be stimulated by the demand of the cell for NADPH. When D-xylose or L-arabinose are used as carbon source, they are metabolized into xylulose-5-phosphate to enter the glycolysis. Thus, no NADPH is formed; however, the cell still needs NADPH for other biochemical reactions (Witteveen et al. 1989; Peleato et al. 1991; David et al. 2005). Consequently, the cell might activate the PPP to produce more NADPH. The total theoretical need for NADPH of a fungal cell has been modelized for *C. utilis* (Bruinenberg et al. 1983; Bruinenberg 1986). With such a model, it is possible to estimate the demand of NADPH based on the carbon and nitrogen source available and to estimate whether the flow towards the PPP will be increased or decreased under these conditions. Notably, stoichiometric models have been established for *A. niger* and *Penicillium chrysogenum* (Jorgensen et al. 1995; David et al. 2003; Melzer et al. 2007).

In *A. niger*, genes of the PPP are upregulated during growth on D-xylose as a sole carbon source (Battaglia et al. 2014). In this work, the expression of PPP genes was compared on the one hand between an *xlnR* deletion strain and the wild-type grown on D-xylose and on the other hand between an *araR* deletion strain and the wild-type grown on L-arabinose.

XlnR is the transcriptional activator of the xylanolytic and cellulolytic system (van Peij et al. 1998). It notably also controls the pentose catabolic pathway (Hasper et al. 2000). The D-xylose catabolic genes are expressed in response to the presence of D-xylose. However, this induction by D-xylose does not impact the transcript level of *xlnR* (Mach-Aigner et al. 2012). *AraR* encodes the transcriptional activator of the L-arabinose catabolic system (de Groot et al. 2007).

Analysis of gene expression was first performed with RNA microarray. In the *araR* deletion strain grown on L-arabinose, seven genes coding for PPP enzymes showed significantly reduced expression compared to the wild type. In the *xlnR* deletion strain grown on D-xylose, the expression of a transaldolase (*talB*) and a transketolase (*tktB*) decreased significantly and, however, were not affected in the *araR* deletion strain. Only one gene was significantly altered in the *xlnR* and the *araR* deletion strain: the RPI-encoding gene *rpiA.* Based on these initial microarray results, some genes were selected for analysis by Northern blot that confirmed the results. Additionally, a Northern blot was performed with RNA from a double deletion *araR/xlnR* strain cultivated on D-xylose or L-arabinose. In this strain, the expression of five PPP genes was reduced on L-arabinose and of four genes on D-xylose. A decrease in the expression of *rpiA* and *talB* was observed in the tested conditions in comparison to the wild type (Battaglia et al. 2014).

The expression of the PPP genes was also analyzed in a strain without D-xylulose kinase activity (*xki*A1 mutant) implicating that D-xylulose cannot be converted in D-xylulose-5-phosphate. Thus, genes, which are induced by D-xylulose-5-phosphate, are expected to show reduced expression, and genes induced by D-xylose or L-arabinose (such as the genes regulated by XlnR and AraR) are expected to show increased expression. Two genes of the PPP had increased levels in the mutant strain, i.e., *talB* in cultures grown on D-xylose and *rpiA* in cultures grown on L-arabinose. The other PPP genes showed reduced or similar expression levels (Battaglia et al. 2014).

Furthermore, Battaglia et al. searched 1kb upstream regions of genes coding for enzymes involved in the pentose catabolic pathway and in the PPP for conserved elements (Battaglia et al. 2014). Six different motifs, of which none was referenced in the JASPAR database, were identified. As these motifs are present in the 1kb upstream region of genes regulated by AraR, they can be considered as putative binding sites for AraR. Further, the consensus sequence of the XlnR DNA-binding site (GGCTA (AG)) was searched in the same set of upstream regions of genes coding for enzymes involved in the pentose catabolic pathway and in the PPP and genes regulated by XlnR. The XlnR DNA-binding site was found in upstream regions of its known target genes and additionally in those of six PPP genes, including *talB* and *rpiA*. This result suggests a regulatory function of XlnR for *talB* and *rpiA*.

### Crosslink between carbon utilization and nitrogen source

Trehalose is a sugar metabolized among others by fungi, plants, and bacteria and has been reported as an environmental stress protectant (Argüelles 1994; Thevelein 1996; Argüelles 2000; Tapia et al. 2015; Magalhães et al. 2018). However, several researchers demonstrated that the role of trehalose in the response to environmental stress might not be linked to trehalose *per se* but to the activation of the environmental stress response by trehalose (Winderickx et al. 1996; Gibney et al. 2015). Trehalose is synthetized by two consecutively acting enzymes: the trehalose-6-phosphate synthase TPS1 and the trehalose-6-phosphate phosphatase. In *S. cerevisiae* the deletion of the *tps1* gene leads to a growth inhibition in presence of glucose. This growth inhibition is associated with an increased hexokinase activity leading to an increased flux into glycolysis, depletion of ATP, and accumulation of the sugar phosphate intermediates (van Aelst et al. 1993; Thevelein and Hohmann 1995). It was reported that the deletion of the hexokinase II-encoding gene *hxk2* restores the ability of *tps1* mutants to grow on glucose, suggesting that TPS1 might contribute to the regulation of the hexokinase activity (Hohmann et al. 1993; Gancedo and Flores 2004).

In *Magnaporthe grisea*, it has been demonstrated that the ability of *tps1* mutants to grow on glucose can be restored by the addition of amino acids, suggesting a link between the carbon and the nitrogen metabolism (Foster et al. 2003). This hypothesis was further tested by an investigation of the relationship between TPS1 and the regulation of the glycolysis (Wilson et al. 2007). In this study, a *tps1* deletion strain was compared to a wild-type strain of *M. grisea*. After cultivation, the levels of G6P and F6P were measured in the mycelia. The authors observed significantly higher amounts of sugar phosphates in the *tps1* deletion strain compared to the wild-type when grown on glucose or in a complete medium (5-fold increase of G6P; F6P increased from an undetectable level to 2 mol/g of mycelium) (Wilson et al. 2007). The complete medium contained 1% glucose, 0.2% peptone, 0.1% yeast extract, and 0.1 % amino acids, while the glucose medium contained 10% glucose and 0.6% sodium nitrate. No increase in F6P was observed in the *tps1* deletion strain compared to other conditions. Next Wilson and co-workers deleted the *hxk1* gene in *M. grisea* analogous to the experiments of Hohmann and co-workers carried out in *S. cerevisiae* (Hohmann et al. 1993). The wild-type, the *tps1* deletion strain, the *hxk1* deletion strain, and the double deletion *hxk1/tps1* strain were cultivated on the complete medium and on the medium with glucose using nitrate as nitrogen source, and the hexokinase activity was measured. The levels of G6P were reduced by 4-fold in the *hxk1* deletion strain and the double deletion strain, while it was 14-fold increase in the *tps1* deletion strain (Wilson et al. 2007). The ability to grow on glucose was partially restored in the double deletion strain; however, the growth was still impaired compared to the wild type. This result indicates that in *M. grisea*, the growth phenotype of a *tps1* deletion strain cannot be restored by the deletion of *hxk1*. Consequently, it can be concluded that TPS1 has a different function in *M. grisea* than in yeasts.

Additional growth tests were performed with the *tps1* deletion strain and the wild type using fructose or glucose as carbon source and different nitrogen sources (10 mM each): amino acids, ammonium, nitrite, and nitrate. Interestingly, the deletion strain was able to grow on both carbon sources similar to the wild type in the presence of ammonium, nitrite, and amino acid (except cysteine). In the presence of nitrate, the growth of the deletion strain was impaired with both carbon sources. Thus, the *tps1* mutant can grow on glucose depending on the nitrogen source. Consequently, Wilson and co-workers performed nitrate reductase activity assays of the *tps1* deletion strain and the wild-type cultivated under different nitrogen conditions. In the wild type, the nitrate reductase activity was increased when grown on nitrate compared to growth on ammonium, whereas in the *tps1* mutant, no activity was detectable under both conditions. Thus, the authors concluded that the inability of *M. grisea* mutants to grow on glucose is not linked to glycolytic misregulation but to the inability to use nitrate (Wilson et al. 2007).

The hypothesis suggested by the authors is that the connection between nitrate utilization and sugar metabolism is the availability of NADPH generated in the OPPP by the G6PDH. Growth on nitrate would increase the hexokinase activity and the NADPH levels in a wild type, while in a *tps1* deletion strain, nitrate would reduce G6PDH activity and NADPH production. Additionally, gene expression analysis showed that the *tps1* mutant expresses NMR1 (nitrogen metabolite repressor 1) when cultivated in the presence of nitrate and that the expression of the nitrite and nitrate reductase-encoding genes is reduced (Wilson et al. 2007). The proposed mechanism is that TPS1 derepresses genes involved in nitrate utilization via NMR1. The extracellular nitrate signal might be perceived via a nitrogen sensing mechanism or through the increased levels of G6P resulting from the increased activity of the hexokinase. Wilson and co-workers concluded that TPS1 might integrate carbon and nitrogen metabolism through G6P sensing, increased NADPH production, and induced expression of genes associated with nitrate utilization (Wilson et al. 2007).

### A network of transcription factors regulate the PPP

The previous examples show that transcriptional regulation is essential for cell adaptation to environmental conditions. Many transcription factors are involved in the regulation of the PPP, and their activation relies on environmental factors such as the respective carbon and/or nitrogen source. Moreover, the PPP is interconnected with the glycolysis and other pathways such as the shikimate pathway. Thus, understanding how transcription factors control the distribution of metabolic fluxes under a given condition is critical to understand the overall regulation of metabolism at the cellular level. This question has been mainly investigated by studying the changes in transcripts levels or/and in protein abundances. However, these parameters do not necessarily allow to predict changes in a cell phenotype or corresponding activities. A study on 119 transcription factors (TFs) performed in *S. cerevisiae* under five growth conditions tackled this gap (Fendt et al. 2010). Basically, this study linked growth conditions to changes in the metabolic flux and to modifications in the expression of TFs. Among 275 genes annotated as “transcriptional regulatory active” in the yeast genome database, the authors selected 119 TFs either related to metabolism or stress response. Then, single mutant strains of these 119 TFs were generated, and the strains were cultivated under five conditions: glucose, galactose, glucose combined with low pH, glucose combined with urea as nitrogen source, and glucose combined with high osmolarity. The chosen conditions cover two common stress factors, reduced and maximal carbon catabolite repression, and two nitrogen sources (urea versus ammonium sulfate). Cultivation was performed with 20% 13C-labeled glucose or galactose, and then the transcriptome (by next-generation sequencing), the metabolome (by GC-MS), and the proteome (nano LC-MS/MS) were determined (Fendt et al. 2010). Fendt and co-workers evaluated six flux ratios corresponding to (i) gluconeogenesis, (ii) glycine production through C1 metabolism, (iii) transport of mitochondrial oxaloacetate into the cytosol, (iv) the relative contribution of glycolysis versus the PPP, (v) the relative contribution of the backward flux from glycine to serine versus the forward flux from 3-phosphoglycerate to serine, and (vi) the relative contribution of the respiratory tricarboxylic acid (TCA) cycle flux versus the replenishment of the biosynthetic precursor. Only three mutants of the 119 mutants exhibited an altered pathway usage of glycolysis versus the PPP. This alteration on the fluxes did not correlate with the specific growth rates; thus, the mutated TFs are responsible for the observed changes. Among all the tested transcription factors, 23 were affected by at least one tested growth condition. All these 23 are involved in controlling the TCA cycle, of which only two regulate the TCA cycle and the PPP, i.e., the retrograde regulation transcription factor 1 and 3 (Rtg1 and Rtg3) (Fendt et al. 2010). Rtg1 and Rtg3 are known as regulators of the mitochondrial retrograde response (Liao and Butow 1993; Chelstowska and Butow 1995).

While 55 tested TFs have at least one target in glycolysis or the PPP, only two altered the flux distribution between these two pathways, namely, PHO2 and BAS1. Mutants of these TFs exhibited an increase of several glycolysis enzymes; however, this did not alter the relative usage of glycolysis versus the PPP (Fendt et al. 2010). PHO2 is a phosphate metabolism homeobox transcription factor regulating genes involved in phosphate metabolism, and BAS1 is a myb-related transcription factor regulating basal and induced expression of genes of the purine and histidine biosynthetic pathways (Arndt et al. 1987; Berben et al. 1988; Lenburg and O’Shea 1996).

These data demonstrate the robustness of the regulation of the PPP and central metabolic fluxes in general. Several hypotheses try to explain this phenomenon. The favored hypothesis is that changes in functional fluxes require several transcription factors acting on a number of target genes. Different signaling pathways might transmit environmental signals to these transcription factors. Interestingly, it has been demonstrated that Rtg1 and Rtg3 are controlled by TOR (target of rapamycine)-mediated control in response to glutamine in *S. cerevisiae*, suggesting an integration of nitrogen and carbon metabolism by the TOR protein (Crespo et al. 2002). Figure 2 provides a schematic drawing of this regulatory cascade. Additionally, it allows a comparison of the nitrogen-related regulation of the PPP between *Aspergillus* and *Saccharomyces* and suggests a hypothesis about the nature of the *nir* product.

## Engineering the PPP in industrially relevant fungi

Fungi have a broad range of applications in biotechnologies: production of food and beverages as well as biofuels, chemicals, enzymes, and also polysaccharides, antibiotics, and medicines (Demain 2014; Katz and Baltz 2016; Meyer et al. 2016; Silber et al. 2016). Fungi used in the biotechnological industry mainly comprise yeasts and filamentous fungi. They produce a vast array of secondary metabolites and an extensive catalogue of proteins, specifically enzymes. The mostly used yeast in the industry is *S. cerevisiae*, while the mostly used filamentous fungi are *Aspergillus* ssp., *Penicillium* ssp., and *T. reesei*. The genomes of industrially used filamentous fungi, such as *A. niger*, *P. chrysogenum*, or *T. reesei*, have been sequenced, which might facilitate the understanding of the PPP and its regulation in these strains (Pel et al. 2007; Martinez et al. 2008; van den Berg et al. 2008).

Another industrially applied fungal family worth to be mentioned is the C1 expression platform. The company Dyadic developed this fungal expression platform from *Myceliophthora thermophila*. The C1 technology is a “robust and versatile fungal expression system for gene discovery, development, expression and production of enzymes and other proteins” according to Dyadic (Visser et al. 2011). Nevertheless, this technology will not be further discussed in this review because very few information has been made public. For example, no insights in the metabolism of this fungus nor in its PPP are available.

Due to its central role in carbon metabolism, the PPP is one of the main targets for metabolic engineering in fungal biotechnologies. The motivation for engineering the PPP can be seen in three major aims: (i) increasing the resistance of the cells to environmental stress, (ii) rerouting the metabolism towards a specific metabolite of interest, and (iii) increasing the NADPH concentration. This review article addresses these three aims by three studies and highlights how these strategies benefit from understanding of the PPP and, in turn, how these reports enlarge the current knowledge.

### Modifications to increase resistance of the cells to stress

Industrial production setups and the requirement for cultivation in bioreactors cause diverse stress factors on fungal strains, such as stirring stress, toxic products synthesized during the process, and oxidative conditions. An example of such a situation is the process for biofuel production. Due to current concerns about climate change, significant efforts are put on developing processes to produce these fuels from renewable feedstocks. A diversity of yeasts can produce alcoholic biofuels, but these alcohols are also toxic for them. Several fungi naturally produce branched-chained alcohol; thus, fungi are a good alternative for developing alcoholic fuel production processes. However, fungi are also sensitive to alcohol resulting in low yields of the processes. Nevertheless, reaching high yields is a prerequisite for cost-effective production and the sustainability of such processes. A strategy to increase the yield is to increase the resistance of the cell to the toxic product (Mukhopadhyay 2015). Modifications of the PPP can help to overcome this challenge.

An example is the research work performed in *S. cerevisiae* to increase its tolerance to isobutanol (Kuroda et al. 2019). As a starting point, the research team studied the BY4741 gene deletion library (Winzeler et al. 1999; Giaever et al. 2002) to identify deletions causing changes in cell growth using medium containing isobutanol. Based on these data, cultivations were performed to confirm an increased sensitivity or increased tolerance of the strains to isobutanol and ethanol. Three strains with a unique phenotype were identified: they were more sensitive to isobutanol, but not to ethanol than the wild type. These strains had deletions of 6PGDH (GND1 in *Saccharomyces*), ZWF1, and the Na+/H+ antiporter NHA1. Further experiments demonstrated that strains with a deletion of GND1 or ZWF1 (NHA1 deletion strains were not tested) have higher sensitivity to C4-C6 alcohols regardless of their branching (Kuroda et al. 2019).

GND1 and ZWF1, respectively, code for the 6PGDH and the G6PDH, two enzymes of the PPP that catalyze reactions generating NADPH. NADPH/NADP+ ratios were lower in the ZWF1 deletion strain and unchanged in the GND1 deletion strain compared to the wild type. Kuroda and co-workers also tested the isobutanol-specific sensitivity of deletion strains lacking an enzyme of the PPP that is not involved in NADPH generating reactions, such as TKL1 or RPE1. The single deletion strains for TKL1 and RPE1 had an isobutanol-specific sensitivity at 1.4% isobutanol similar to the ZWF1 and the GND1 deletion strains (Kuroda et al. 2019). Thus, the reduced tolerance to isobutanol cannot be linked only to a reduced amount of NADPH as often stated in studies on yeast tolerance to environmental stress (Gorsich et al. 2006; Matsufuji et al. 2008). The role of the PPP concerning the sensitivity to isobutanol cannot be limited only to the provision of NADPH. This is consistent with the fact that the role of the PPP in the oxidative stress response is also not limited to NADPH production (Park et al. 2019). The work from Kuroda and co-workers provides a link between sensitivity to environmental stress and the deletion of enzymes of the PPP (Park et al. 2019). However, no mechanism was proposed to explain this phenomenon. It rather demonstrated the complexity of the regulation networks involving the PPP. A lack of an enzyme of the PPP might impact more than just the product of the catalyzed reaction.

### Modification to reroute metabolism towards a metabolite of interest

Besides the natural ability of fungi to produce a wide range of useful molecules, they can use a diversity of carbon and nitrogen sources, an asset in the context of focusing on sustainable nutrient resources. Moreover, some fungi and yeasts are also classified as safe, making them ideal industrial production hosts. Many of these microorganisms can be engineered to gain additional functions. In the following example, *S. cerevisiae* has been engineered to produce shinorine (Park et al. 2019).

Shinorine is a mycosporine, or mycosporine-like amino acid, used as sunscreen material in biobased products. The demand for these products increases, but the production yield of the natural producers (cyanobacteria and red algae, e.g., *Porphyra umbilicalis*) is low. The developed strategy used knowledge about the PPP for several purposes. Briefly, the PPP was modified to increase the formation of the product of interest by directing the cell metabolism towards a specific carbon source, namely, D-xylose, and at the same time, to reduce the formation of side products. First, shinorine biosynthetic genes from cyanobacterium *Nostoc punctiform* were introduced into *S. cerevisiae* leading to a strain able to synthesize shinorine at a concentration of 0.67 mg/l at best. To enhance the production yield, it was aimed to increase the formation of the PPP intermediate S7P; S7P is a precursor in the shinorine synthesis pathway. The usage of D-xylose was supposed to increase the S7P production because D-xylose cannot enter glycolysis; thus, its use as carbon source allows redirecting carbons towards the PPP and avoids competition with the glycolysis. The genomic introduction of a gene from *Scheffersomyces stipitis* enables the reduction and metabolism of D-xylose. The obtained strain reached concentrations of 17.99 mg/l shinorine in a medium with 18 g/l D-xylose and 2 g/l glucose (Park et al. 2019).

To further increase the shinorine production, the authors deleted TAL1 in order to prevent the conversion from S7P to G3P. The obtained strain showed a severe growth defect in rich D-xylose medium, presumably because TAL1 is necessary for D-xylose assimilation. Nevertheless, by reducing the amount of D-xylose in the medium, a yield of shinorine of 21.9 mg/l was reached. To compensate for the deletion of TAL1 and to enhance the flux through the PPP, the sin three binding protein 5 (STB5) and the TKL1 were overexpressed. STB5 is a transcription factor involved in response to oxidative stress. In oxidative conditions, it activates PPP genes, including ZWF1, GND1, and TKL1, leading to increased NADPH synthesis. TKL1 was chosen due to its involvement in D-xylose metabolism. The effects of overexpression of the single genes were compared to an empty vector strain and a strain with overexpression of both STB5 and TKL1. The double overexpression strain had increased D-xylose consumption and shinorine production. Finally, by medium optimization, a shinorine production of 31.0 mg/l was reached in a medium containing 8 g/l of D-xylose and 12 g/l of glucose with the latter strain. In this study, the understanding of the PPP was successfully employed to increase the production of a compound of interest. The impact of the carbon source and the known function of TFs were also taken into account. The consequence of the deletion of TAL1 emphasizes the limitations of the strategy to modify the expression of a particular enzyme of the PPP without offering any compensation. The involvement of the PPP in multiple regulation networks makes outcomes of such a modification approach hard to predict.

### Modifications towards NADPH production

The availability of NADPH is a determining factor for many biochemical reactions, and the PPP is the primary source of NADPH. Consequently, increasing the flux through the PPP could be a strategy to generate more NADPH and, thus, can increase the yield of biochemical metabolites. Poulsen et al. have used a strategy to engineer the PPP in order to increase NADPH concentration in *A. niger* (Poulsen et al. 2005).

In this study, three enzymes of the PPP were overexpressed, i.e., the G6PDH, the 6PGDH, and the TKL. The wild-type and the engineered strains were cultured in bioreactors with ammonium or nitrate as the nitrogen source. The data were analyzed by multivariate data analysis using the following variables: G6PDH, 6PGDH, TKL, mannitol-1-phosphate dehydrogenase, S7P, dihydroxyacetone phosphate, Xu5P, F6P, pyruvate, R5P, G3P, 6PG, NADP, NADPH, NADH, i-erythritol, i-arabitol, i-mannitol, i-arabitol, e-trehalose, oxalate, NADH, aldolase (EC 4.1.2.13), TAL (EC 2.2.1.2), PGI, glycerol dehydrogenase (EC 1.1.1.156), G6P, ADP, AMP, NAD, catabolic reduction charge, i-glycerol, i-trehalose, glycerol, e-erythritol, and e-mannitol (i-, intracellular; e-, extracellular). The primary outcome of the study was that the strain overproducing 6PGDH is the only one with an increased NADPH concentration, while no effect of the overproduction of G6PDH and TKL was observed. The increased NADPH concentration did not affect the overall strain physiology: the specific growth rate and the spore formation were not changed compared to the wild type.

These findings suggest that engineering to increase NADPH concentration can be a relevant target to increase the synthesis of a biochemical metabolite. However, the latter was not demonstrated in this work. Indeed, no metabolite of which the biosynthesis requires NADPH was investigated.

## Conclusion

The current understanding of the PPP suggests a complex regulatory control of the PPP and a role of this pathway beyond the provision of NADPH. The studies discussed in this review suggest that nutrient sources such as carbon, nitrogen, and glutamine impact the regulation of the PPP and might interact in their regulation of the PPP. The current knowledge suggests a common regulation through the TOR pathway which controls a complex network of TFs. However, experiments are needed to confirm this hypothesis and to find the missing links between the regulatory impacts of carbon sources, nitrogen sources, glutamine, and the TFs involved.

The metabolic engineering approaches described in the last part of this review demonstrate the potential of engineering the PPP for improvement of biotechnological processes. They also revealed how much is still to learn about the PPP and the difficulty to optimize the production of a metabolite without a complete understanding of the metabolic regulations involved. Fungi have already been in the past significant providers of industrial or medical products, even despite the lack of a full comprehension of their metabolism. Improved knowledge and according modification of the PPP could start a new era in industrial processes and lead to further increase in yields of manufacturing processes.

Finally, solving the regulation of the PPP in fungi might improve insights in the PPP of higher eukaryotes as humans. The PPP is studied in humans, especially in relation to cancer and diabetes, where enzymes of the PPP are targets for possible treatments. The study of fungal cells is easier to carry out and poses less ethical and safety concerns than the work on human or animal cells. Obtained insights from fungi could lead to a progress in the understanding of relevant issues related to the PPP in humans.

## Abbreviations

6PG: 6-phosphogluconate
6PGDH: 6-phosphogluconate dehydrogenase
6PGL: 6-phosphogluconolactonase
CHP: cumene-hydroperoxide
*cnx*: gene coding for an activator of the enzyme-like protein involved in molybdopterin biosynthesis
cnx-: *cnx*-deficient mutant strain
DHAP: dihydroxyacetone phosphate
DPN: diphosphopyridine nucleotide
E4P: erythrose-4-phosphate
F6P: fructose-6-phosphate
G3P: glyceraldehyde-3-phosphate
G6P: glucose-6-phosphate
G6PDH: glucose-6-phosphate dehydrogenase
GND1: 6-phosphogluconatedehydrogenase in *Saccharomyces* (same as G6PDH)
GPI: glucose-6-phosphate isomerase
HXK1: hexokinase 1
LC-MS/MS: liquid chromatography coupled with tandem mass spectrometry
NAD+: nicotinamide adenine dinucleotide (same as DPN)
NADH: nicotinamide adenine dinucleotide + hydrogen
NADP+: nicotinamide adenine dinucleotide phosphate (same as TPN)
NADPH: nicotinamide adenine dinucleotide phosphate + hydrogen
NHA1: Na+/H+ antiporter 1
*nia*D: gene coding for the nitrate reductase
*nia*D-: *nia*D*-*deficient mutant strain
*nii*A: gene coding for the nitrite reductase
*nii*A-4: *niiA*-deficient mutant strain
*nir*A: gene coding for the transactivator NirA, activates genes associated to nitrate metabolism
*nir*A-1: *nirA*-deficient mutant strain
NMR1: nitrogen metabolism repressor 1
NOPPP: non-oxidative pentose phosphate pathway
OPPP: oxidative pentose phosphate pathway
PHO2: phosphate metabolism 2
PPP: pentose phosphate pathway
R5P: ribose-5-phosphate
RPE: ribulose-5-phosphate 3-epimerase
RPI: ribose-5-phosphate isomerase
*rpiA*: gene coding for the ribose-5-phosphate isomerase A
Rtg1: retrograde regulation transcription factor 1
Rtg3: retrograde regulation transcription factor 3
Ru5P: ribulose-5-phosphate
S7P: sedoheptulose-7-phosphate
SHPK: sedoheptulose kinase
SOL4: suppressor of Los1-1 4
STB5: sin three binding protein 5
TAL: transaldolase
TCA: tricarboxylic acid
TFs: transcription factors
TKL: transketolase
TOR: target of rapamycine
TPI: triose phosphate isomerase
TPS1: trehalose-6-phosphate synthase 1
TPN: triphosphopyridine nucleotide
XlnR: transcriptional activator of the xylanolytic system (in *Aspergillus*)
ZWF1: Zwischenferment (in *Saccharomyces*, same as G6PDH)
